# Supplementary material for: Active state structures of a bistable visual opsin bound to G proteins
Source: Nat Commun. 2024 Oct 16;15:8928. doi: 10.1038/s41467-024-53208-2 (PMC11484933; doi:10.1038/s41467-024-53208-2)
Supplement: Supplementary file 1 — Supplementary Information [file 41467_2024_53208_MOESM1_ESM.pdf]

Supplementary Information for

Active state structures of a bistable visual opsin bound to G proteins

Oliver Tejero[1,2], Filip Pamula[1,‡], Mitsumasa Koyanagi[3,4], Takashi Nagata[5,§], Pavel Afanasyev[6], Ishita Das[7], Xavier Deupi[1,8,9], Mordechai Sheves[7], Akihisa Terakita[3,4], Gebhard F.X. Schertler[1*], Matthew J. Rodrigues[1*], Ching-Ju Tsai[1*]

[1] Laboratory of Biomolecular Research, Department of Biology and Chemistry, Paul Scherrer Institute, 5232 Villigen-PSI, Switzerland.

[2] Department of Biology, ETH Zurich, 8093 Zurich, Switzerland.

[3] Department of Biology, Graduate School of Science, Osaka Metropolitan University, Osaka 558-8585, Japan.

[4] The OMU Advanced Research Institute of Natural Science and Technology, Osaka Metropolitan University, Osaka 558-8585, Japan.

[5] Department of Biology and Geosciences, Graduate School of Science, Osaka City University, Osaka 558-8585, Japan

[6] Cryo-EM Knowledge Hub, ETH Zurich, 8093 Zürich, Switzerland.

[7] Department of Molecular Chemistry and Materials Science, Weizmann Institute of Science, 7610001 Rehovot, Israel.

[8] Condensed Matter Theory Group, Laboratory of Theoretical and Computational Physics, Division of Scientific Computing, Theory and Data, Paul Scherrer Institute, 5232 Villigen-PSI, Switzerland.

[9] Swiss Institute of Bioinformatics (SIB), 1015 Lausanne, Switzerland.

‡ Current address: Department of Molecular Biology and Genetics, 8000 Aarhus University, Aarhus, Denmark.

§ Current address: Institute for Solid State Physics, The University of Tokyo, 5-1-5 Kashiwanoha, Kashiwa, Chiba 277-8581 Japan.

* Corresponding authors.

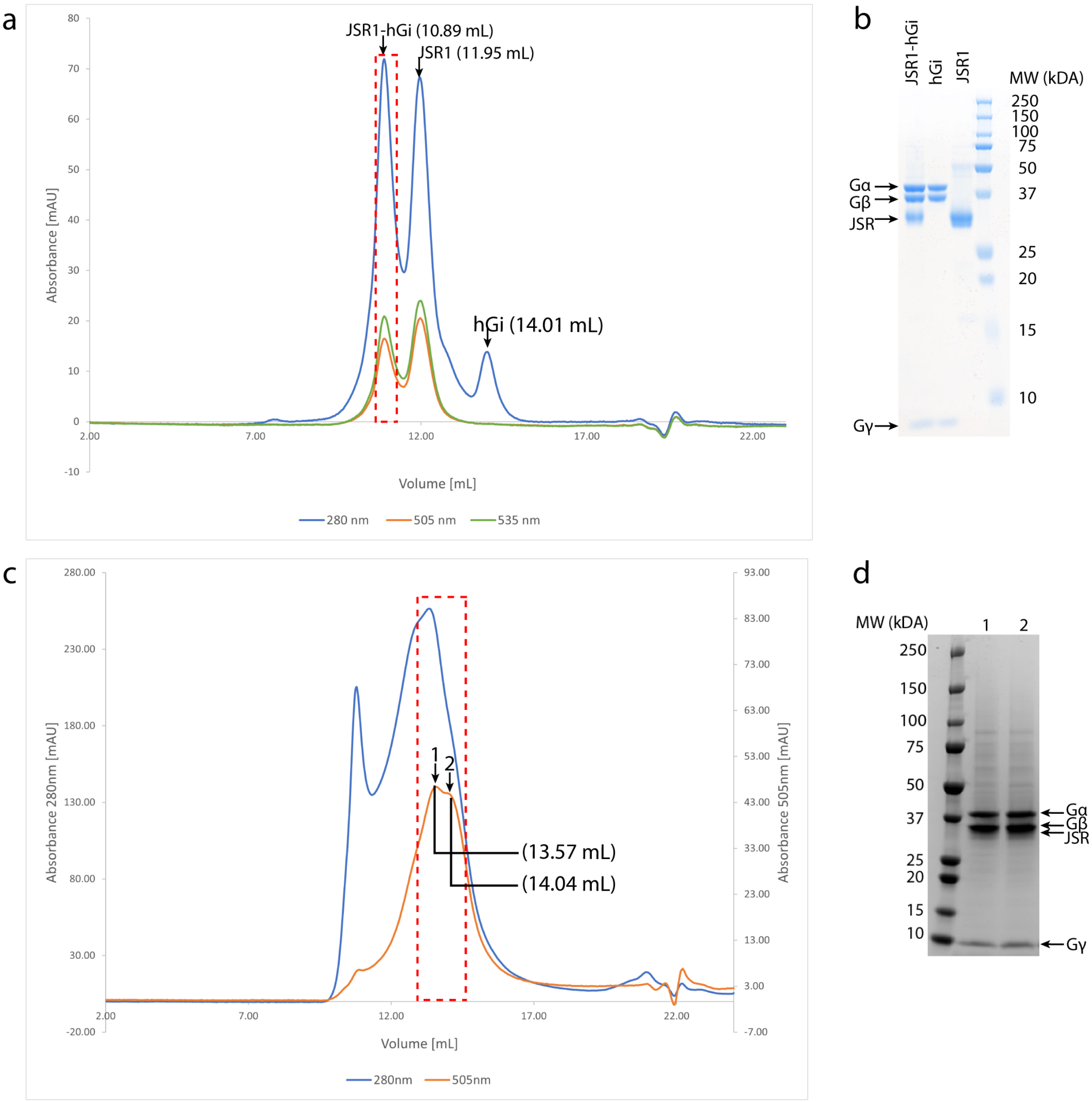

Supplementary Figure 1: SEC and SDS-PAGE of the JSR1-hG$_i$ and JSR1-jsG$_{iq}$ complexes. a: SEC chromatogram of the JSR1-hG$_i$ complex. The size exclusion chromatography was performed using a Superdex 200 Increase 10/300 column. Y-axis shows the absorbance in mAU and x-axis shows the volume in mL. The red dashed line marks the fractions collected for freezing on EM grids. **b:** Coomassie stained SDS-PAGE of the three peaks labeled in A. Individual subunits are labeled on the left side and the molecular weight (MW) is indicated on the right side in kDa. **c:** SEC chromatogram of the JSR1-jsG$_{iq}$ complex. The SEC profile was recoded using a SRT-C 300 10/300 column. Y-axis shows the absorbance in mAU and x-axis shows the volume in mL. The red dashed line marks the fractions collected for freezing on EM grids. **d:** Coomassie stained SDS-PAGE of the two fractions indicated in C. Left side shows the MW in kDa and individual subunits are labeled on the right side. The bands for Gβ and JSR1 overlap.

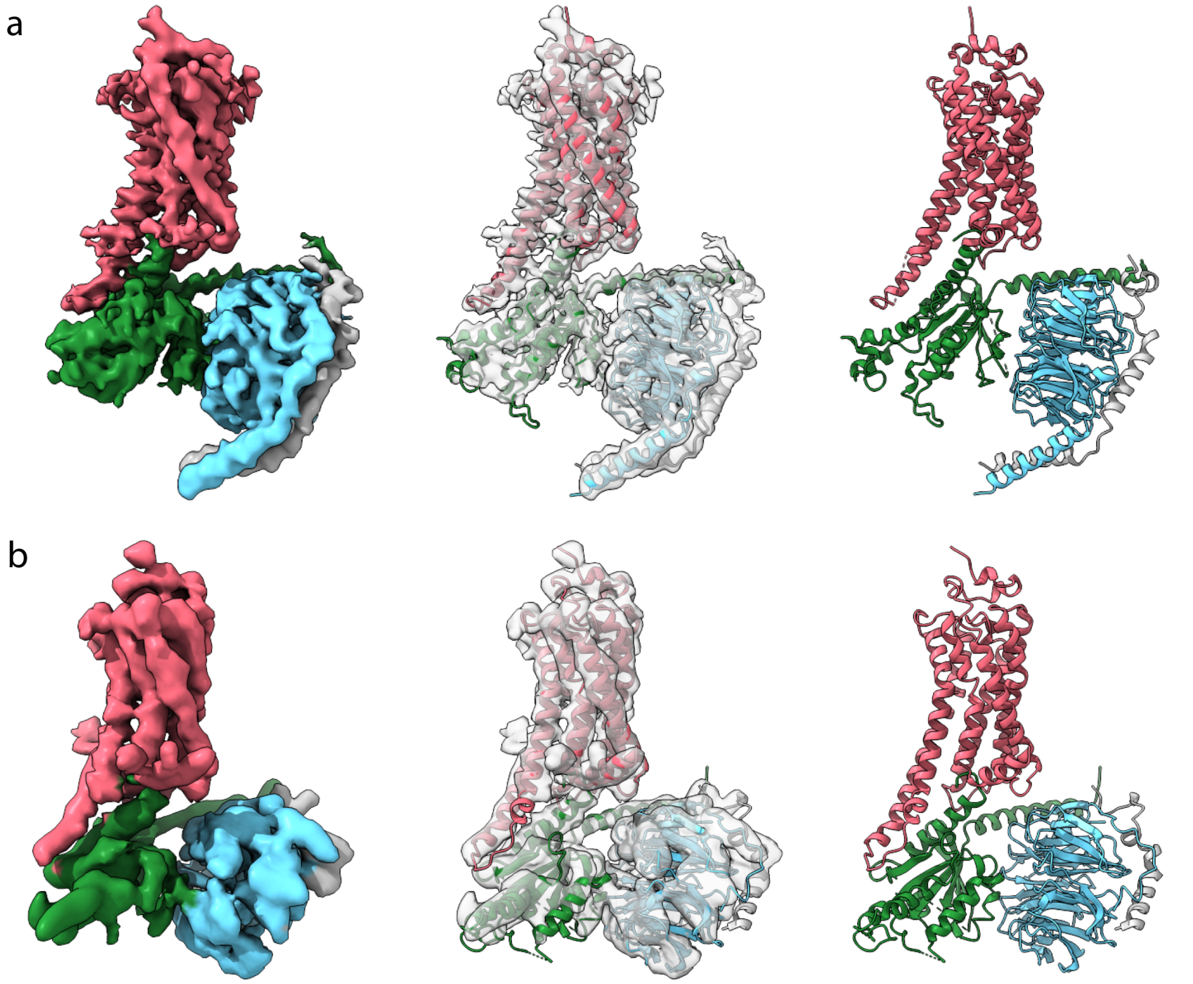

Supplementary Figure 2: Cryo-EM maps and structural model for the JSR1-jsG$_{iq}$_2 complex and the JSR1-hG$_i$ complex. a: Left: JSR1-jsG$_{iq}$_2 cryo-EM map colored by subunit. JSR1, jsGα_{iq}, Gβ and Gγ subunits are shown in salmon, green, cyan and gray, respectively. Middle: Cryo-EM map is shown in gray and transparent with the model of JSR1-jsG$_{iq}$_2 fitted into the map. Subunits of the model are colored in the same way as the map on the left. Right: Model of the JSR1-jsG$_{iq}$_2 complex shown as cartoon. Subunits are colored as in the left cryo-EM map. **b:** Same figures as in A, colored in the same way, for the JSR1-hG$_i$ complex.

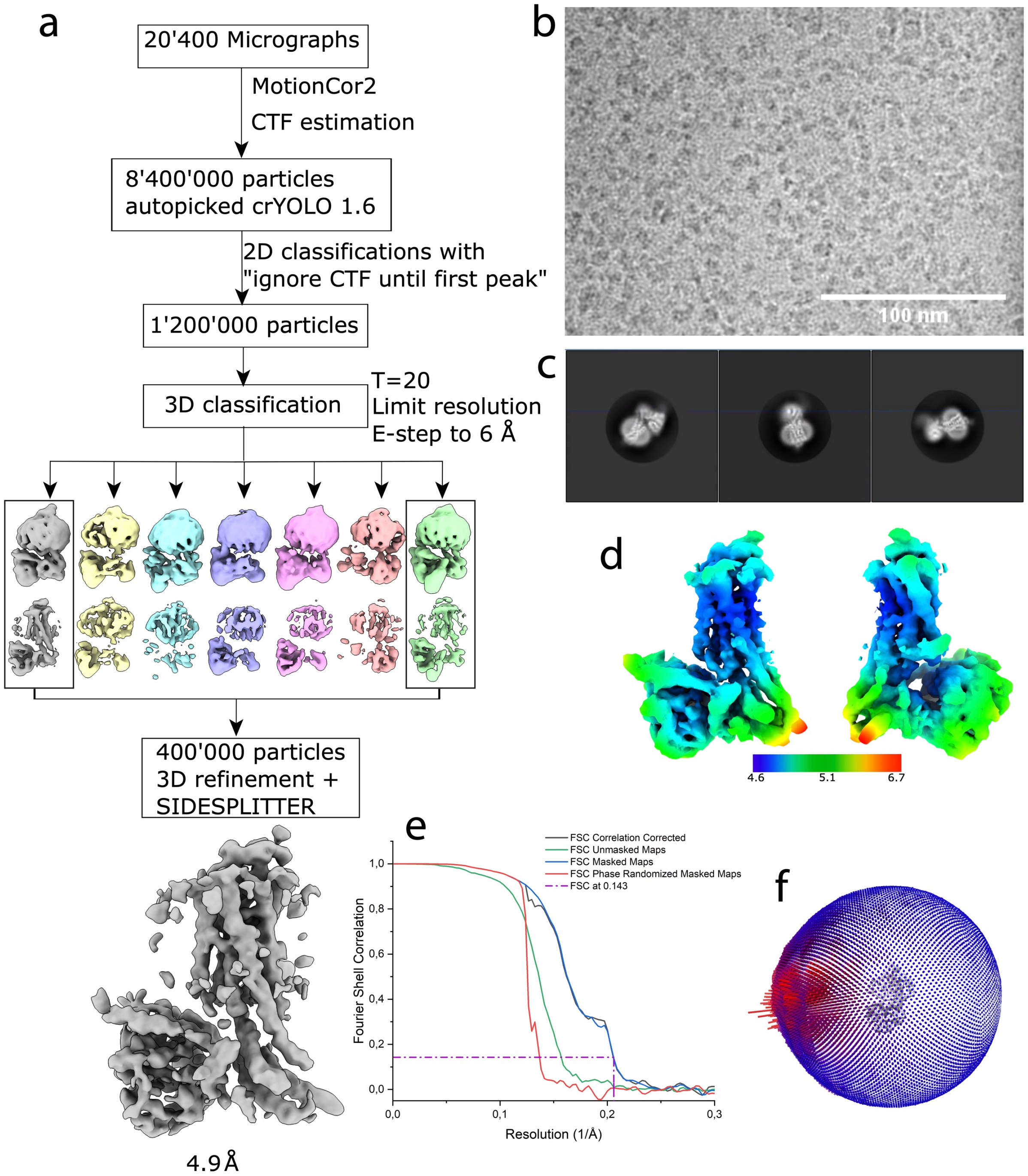

Supplementary Figure 3: Single-particle analysis of the JSR1-hG$_i$ complex. A: Single particle data processing workflow for the JSR1-hG$_i$ complex. **B:** Representative micrograph. **C:** 2D class averages for the JSR1-hG$_i$ complex in different orientations. **D:** 4.9 Å JSR1-hG$_i$ complex electron potential map colored by local resolution values. **E:** FSC plot for the JSR1-hG$_i$ complex at 4.9 Å resolution determined at 0.143 cut-off. **F:** 3D Angular distribution of particles.

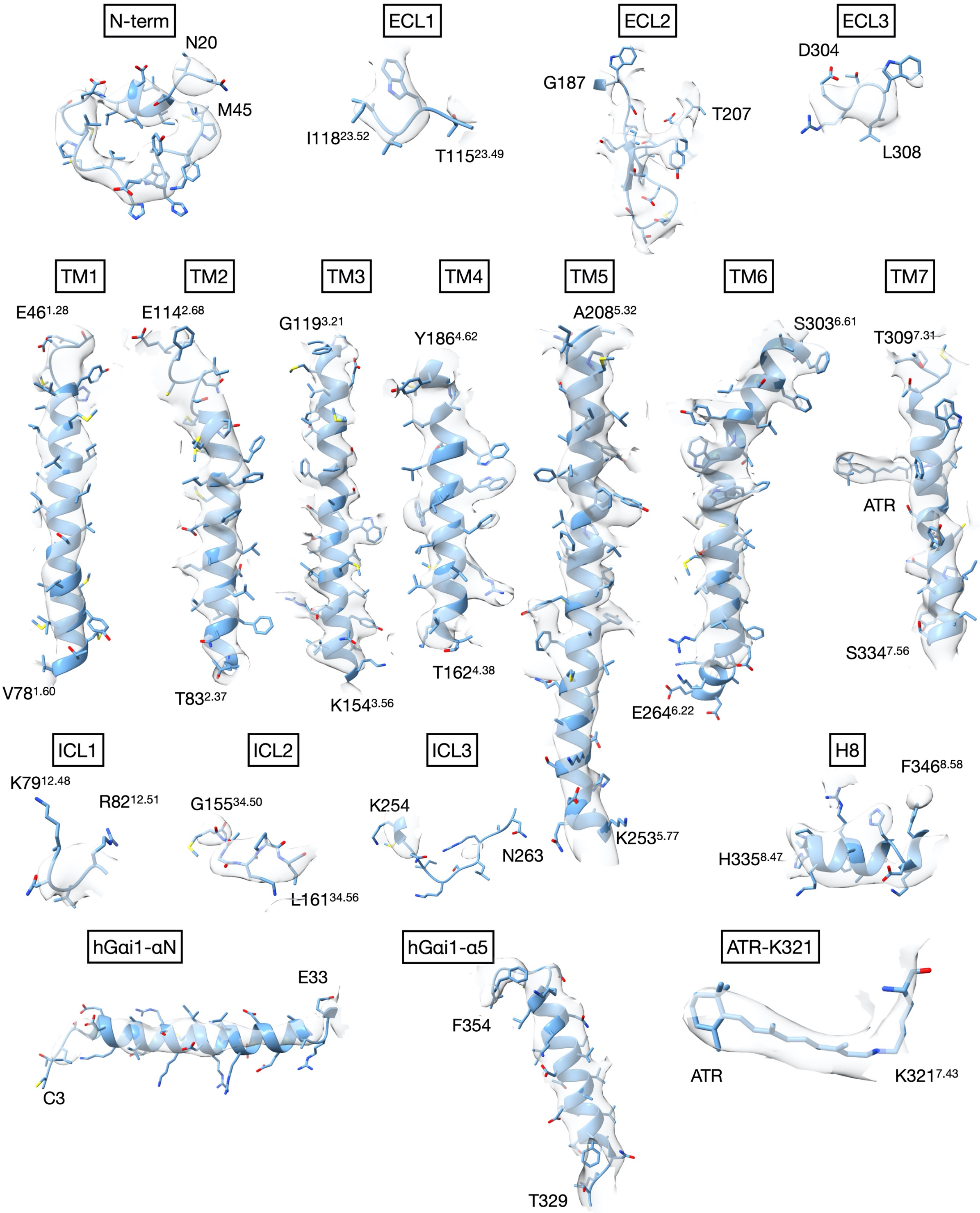

Supplementary Figure 4: Cryo-EM map quality and model fit of the JSR1-hG$_i$ structure. JSR1 is dissected into the N-terminus (N-term), extracellular loops (ECL) 1-3, transmembrane helices (TM) 1-8, helix 8 (H8), and intracellular loops (ICL) 1-3. All-trans retinal (ATR) is shown in two views, one with TM7 and the other with Lys321$^{7.43}$. The αN and α5 helices of hGαi are shown. Protein main chains are displayed in cartoon. Protein side chains and ATR are displayed in stick format with carbon in light blue, nitrogen in dark blue, sulfur in yellow, and oxygen in red.

a

	H5.01	H5.02	H5.03	H5.04	H5.05	H5.06	H5.07	H5.08	H5.09	H5.10	H5.11	H5.12	H5.13	H5.14	H5.15	H5.16	H5.17	H5.18	H5.19	H5.20	H5.21	H5.22	H5.23	H5.24	H5.25	H5.26
Human Gi	T	K	N	V	Q	F	V	F	D	A	V	T	D	V	I	I	K	N	N	L	K	D	C	G	L	F
Human Gq	T	E	N	I	R	F	V	F	A	A	V	K	D	T	I	L	Q	L	N	L	K	E	Y	N	L	V
Hasarius Gq1 visual	T	E	N	I	R	L	V	F	C	A	V	K	D	T	I	L	Q	N	N	L	K	E	C	N	L	V
Hasarius Gq2	T	E	N	I	R	F	V	F	A	A	V	K	D	T	I	L	Q	L	N	L	K	E	Y	N	L	V

b

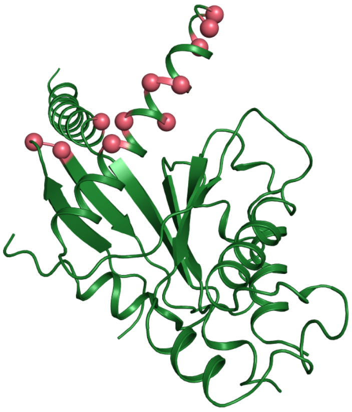

c

Residue number (hGi)	Mutation
31	A→R
193	D→S
194	L→I
337	D→C
340	T→K
342	V→T
344	I→L
345	K→Q
350	D→E
352	G→N
354	F→V

Supplementary Figure 5: Design of chimeric jsGα_{iq} subunit. a: Sequence alignment of the α5 helices of the human Gα_i, human Gα_q, jumping spider visual Gα_{q1} (HaGq1: acc. No. LC799818) and jumping spider Gα_{q2} (HaGq2: acc. No. LC799819). **b:** Human Gα_i structure from our JSR1-jsG$_{iq}$_1 complex. Locations where mutations were introduced are marked as red spheres. **c:** Table with the mutations introduced to make the chimeric jsG$_{iq}$ α subunit using hGα_i as a template.

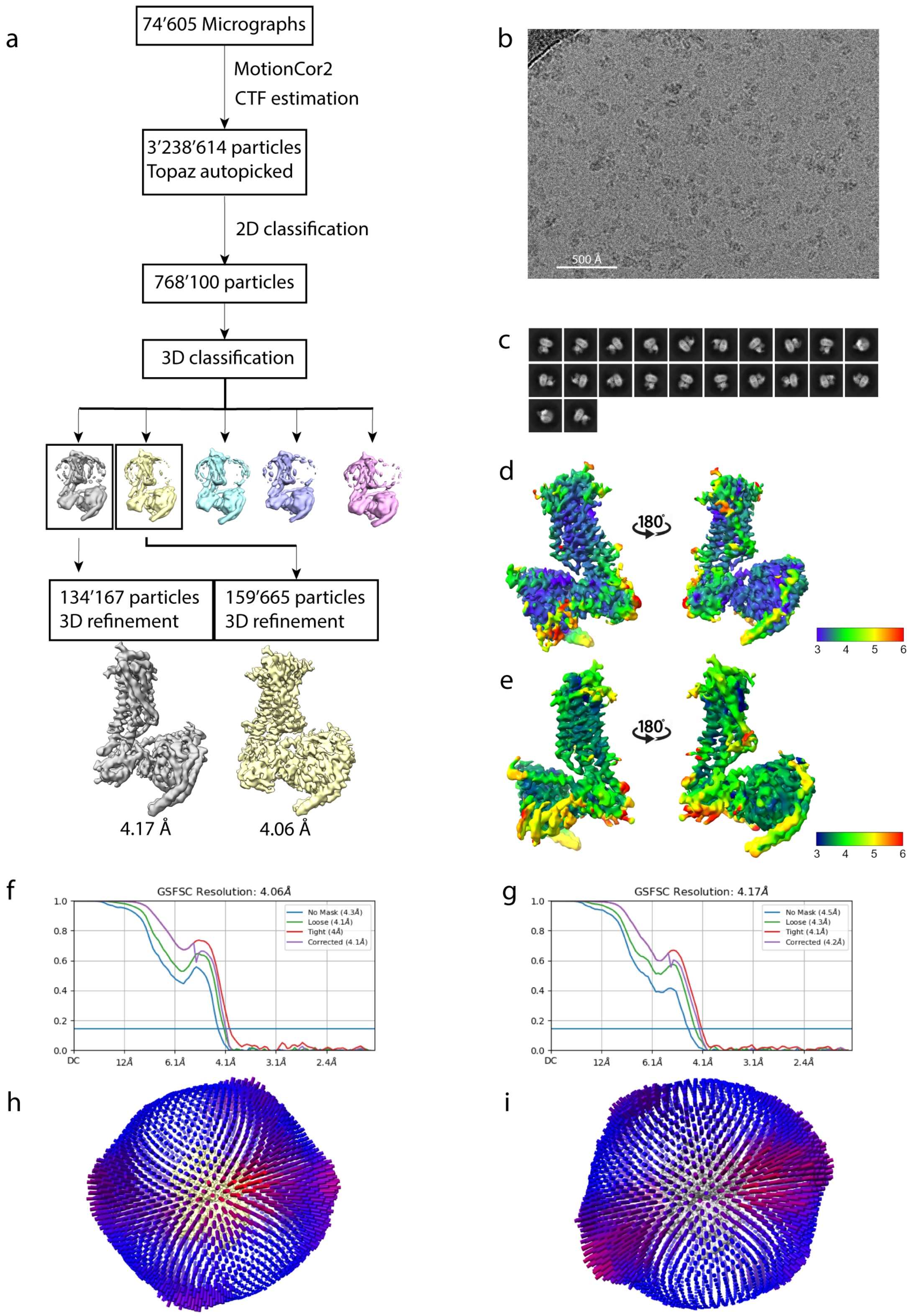

Supplementary Figure 6: Single-particle analysis of the JSR1-jsG_{iq} complex. a: Single particle data processing workflow for the JSR1-jsG_{iq} complex. **b:** A representative micrograph taken at a nominal magnification of 165’000 (0.51 Å/pixel). **c:** Selected 2D class averages for the JSR1-jsG_{iq} complex. **d:** 4.1 Å JSR1-jsG_{iq}_1 complex electron potential map colored by local resolution values (Local Resolution Estimation, cryoSPARC). **e:** 4.2 Å JSR1-jsG_{iq}_2 complex electron potential map colored by local resolution values (Local Resolution Estimation, cryoSPARC). **f:** FSC plot for the JSR1-jsG_{iq}_1 complex at 4.1 Å resolution determined by 0.143 cut-off. **g:** FSC plot for the JSR-jsG_{iq}_2 complex at

4.2 Å resolution determined by 0.143 cut-off. **h:** 3D angular distribution of the JSR1-jsG_{iq}_1 particle set. **I:** 3D angular distribution of the JSR1-jsG_{iq}_2 particle set.

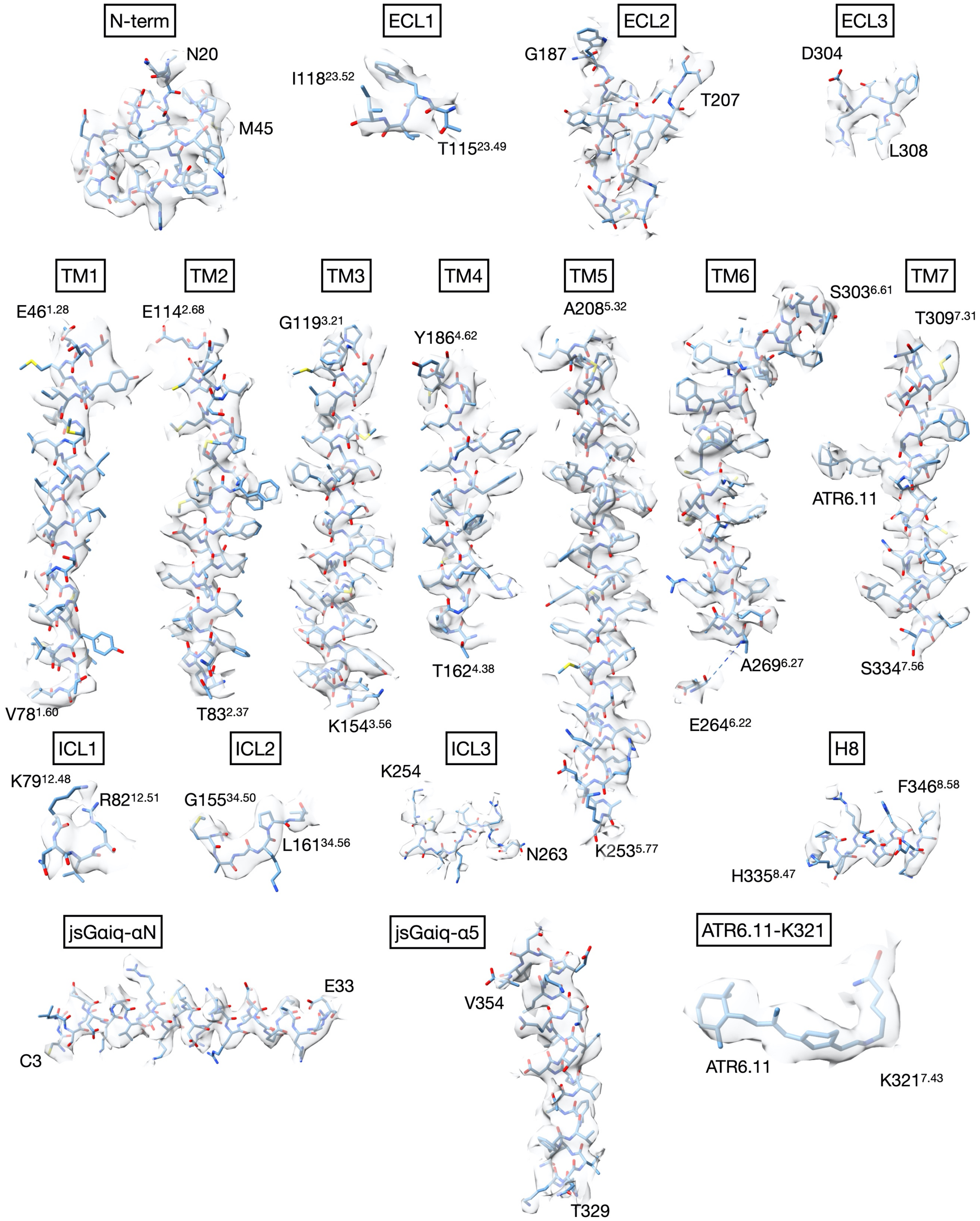

Supplementary Figure 7: Cryo-EM map quality and model fit of the JSR1-jsG$_{iq}$_1 structure. JSR1 is dissected into the N-terminus (N-term), extracellular loops (ECL) 1-3, transmembrane helices (TM) 1-8, helix 8 (H8), and intracellular loops (ICL) 1-3. ATR6.11 is shown in two views, one with TM7 and the other with Lys321. The αN and α5 helices of jsGαiq are shown. Protein residues and ATR6.11 are displayed in stick format with carbon in light blue, nitrogen in dark blue, sulfur in yellow, and oxygen in red.

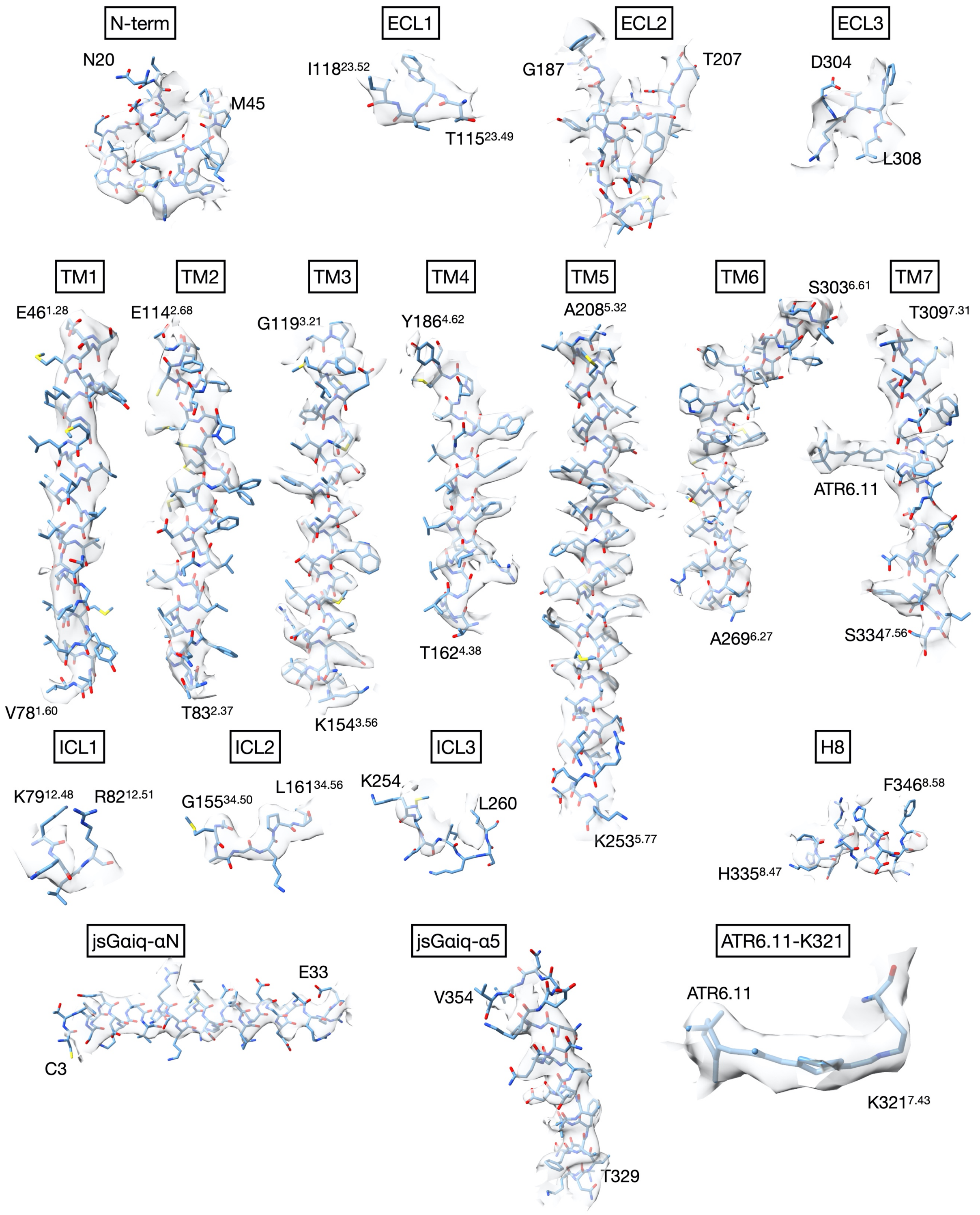

Supplementary Figure 8: Cryo-EM map quality and model fit of the JSR1-jsG$_{iq}$_2 structure. JSR1 is dissected into the N-terminus (N-term), extracellular loops (ECL) 1-3, transmembrane helices (TM) 1-8, helix 8 (H8), and intracellular loops (ICL) 1-3. ATR6.11 is shown in two views, one with TM7 and the other with Lys321. The αN and α5 helices of jsGαiq are shown. Protein residues and ATR6.11 are displayed in stick format with carbon in light blue, nitrogen in dark blue, sulfur in yellow, and oxygen in red.

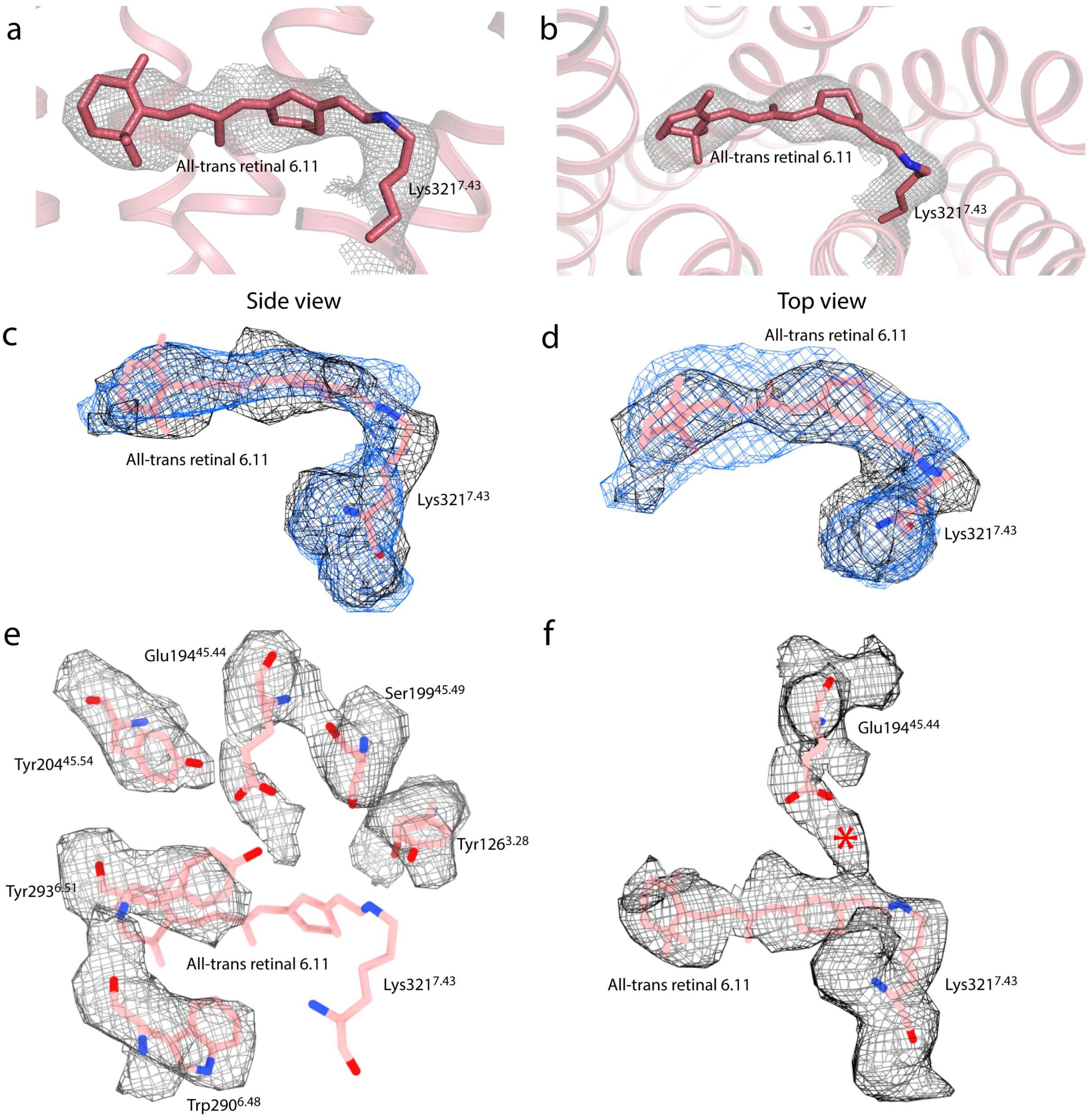

Supplementary Figure 9: All-trans retinal 6.11 with the experimental cryo-EM map and comparison to the all-trans retinal from the JSR1-hG$_i$ complex. a, b: Visualization of all-trans retinal 6.11 from the JSR1-jsG$_{iq}$_1 complex including the experimental cryo-EM map for the Lys321 and all-trans retinal 6.11 shown as a mesh. A shows a side view and B shows a top view from the extracellular side. **c, d:** Overlay of the all-trans retinal 6.11 with the electron density map from the JSR1-jsG$_{iq}$_1 complex (black mesh) and the electron density map of all-trans retinal from the JSR1-hG$_i$ complex (blue mesh). C shows a side view and D shows a top view from the extracellular side. **e:** Residues in the retinal environment shown with the experimental cryo-EM map overlaid as a mesh. **f:** Counterion (Glu194) and retinal with the Schiff base link with the experimental cryo-EM map overlaid. The red asterisk marks a potential solvent binding site.

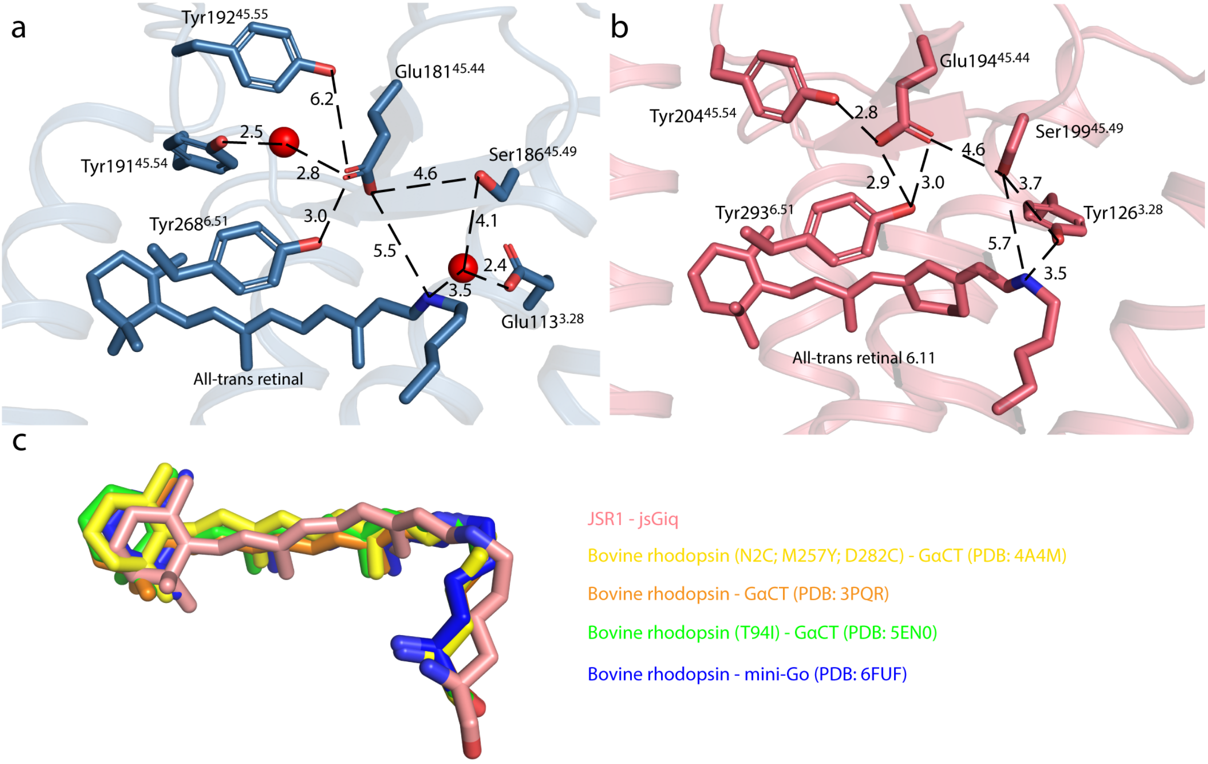

Supplementary Figure 10: Retinal binding site comparison of bovine metarhodopsin-II and active state JSR1. a: Retinal environment of bovine metarhodopsin II (PDB 5EN0). Important residues are shown as sticks. Carbon atoms are colored sky blue, nitrogen atoms blue and oxygen atoms red. Water molecules are depicted as red spheres. **b:** All-trans retinal 6.11 environment of the active JSR1. Corresponding residues from A are shown as sticks for JSR1 and carbon atoms are shown in salmon, nitrogen atoms in blue and oxygen atoms in red. **c:** Overlay of the all-trans retinal 6.11 from our JSR1 structure and all-trans retinal from four bovine metarhodopsin II structures (PDB 4A4M (yellow); 3PQR (orange); 5EN0 (green); 6FUF (blue)).

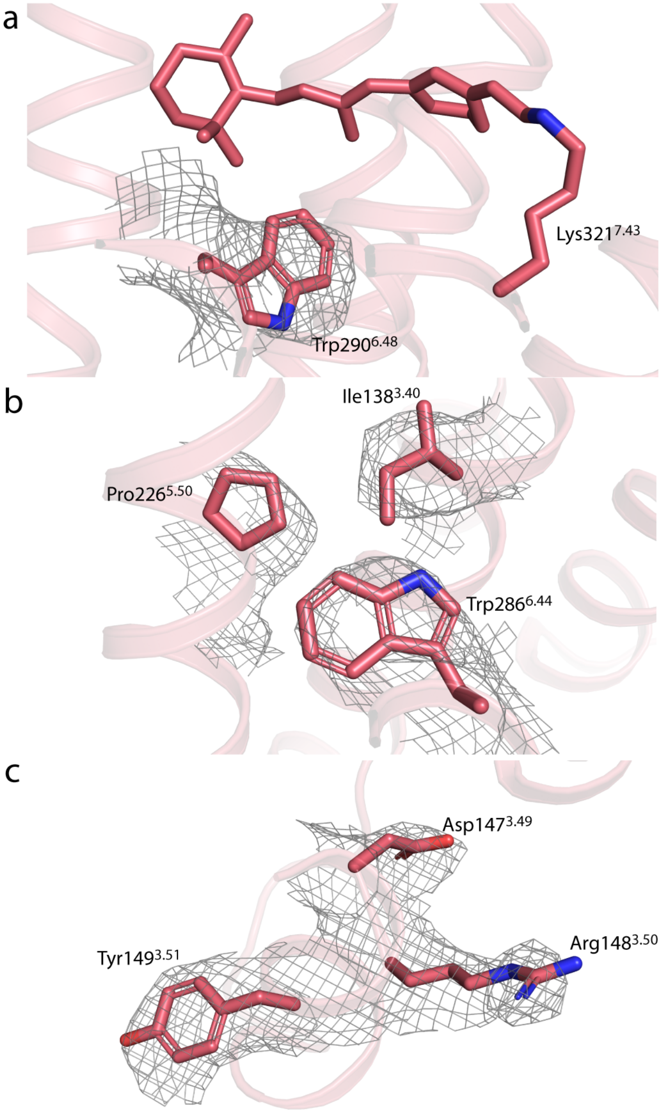

Supplementary Figure 11: Microswitches with experimental cryo-EM map. a: Trp290$^{6.48}$ from the C-W-x-P motif shown in stick representation with the experimental map shown as a mesh. **b:** P-I-F motif with side chains shown as sticks and the experimental map shown as a mesh. **c:** D-R-Y motif with side chains shown as sticks and the experimental map shown as a mesh.

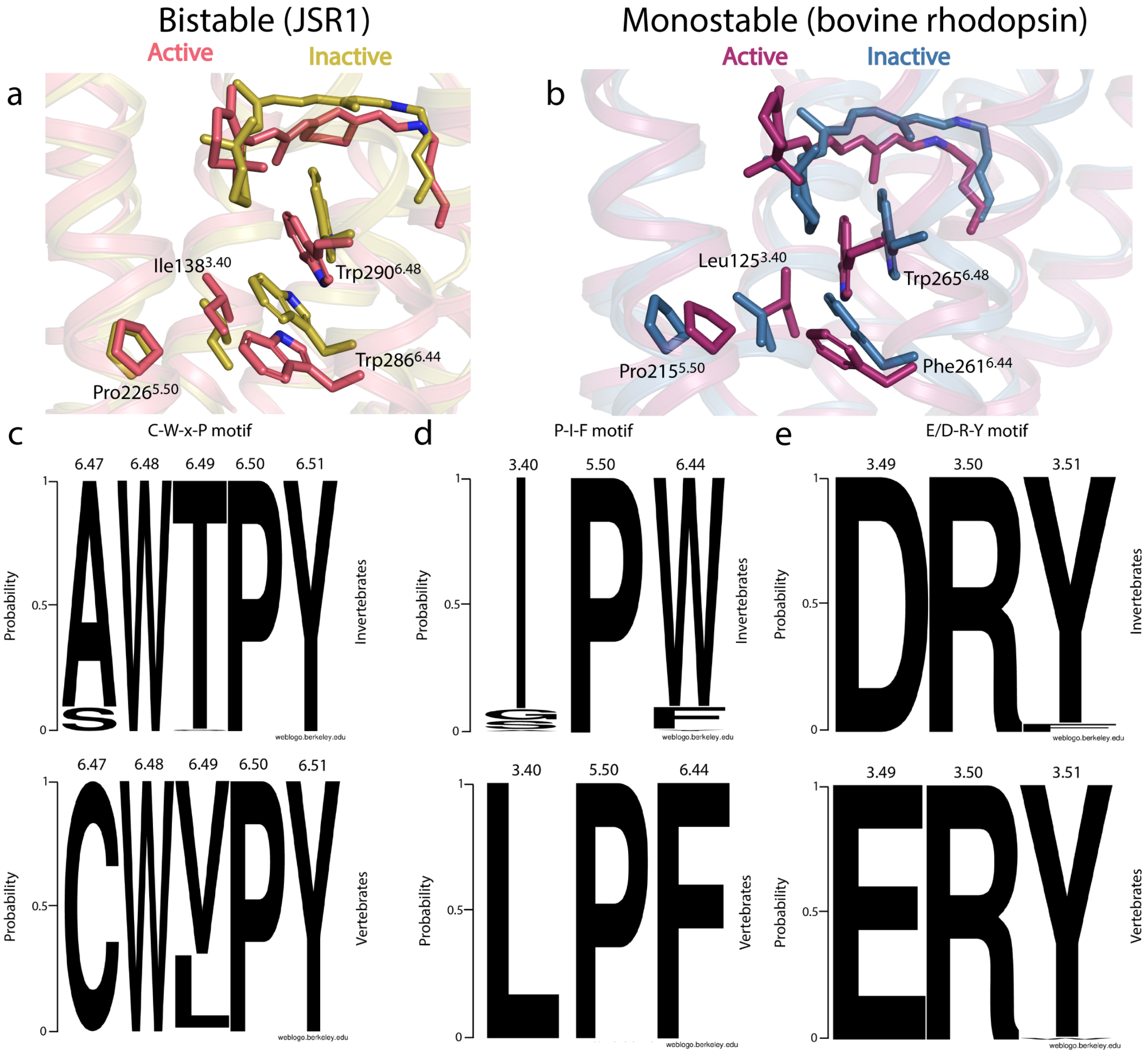

Supplementary Figure 12: Conformational changes upon activation and sequence conservation at the microswitches. a: Conformational changes from the retinal isomerization to the P-I-F motif, leading to the outward movement of TM6. Inactive state JSR1 is shown is olive (PDB 6I9K) and active state JSR1 is shown in salmon. **b:** Conformational changes from the retinal isomerization to the P-I-F motif, leading to the outward movement of TM6. Inactive state bovine rhodopsin (PDB 1GZM) is shown in blue and active state bovine metarhodopsin II (PDB 5EN0) is shown in purple. **c:** Sequence conservation of the C-W-x-P motif in invertebrate rhodopsins (top) and vertebrate rhodopsins (bottom). **d:** Sequence conservation of the P-I-F motif in invertebrate rhodopsins (top) and vertebrate rhodopsins (bottom). **e:** Sequence conservation of the E/D-R-Y motif in invertebrate rhodopsins (top) and vertebrate rhodopsins (bottom).

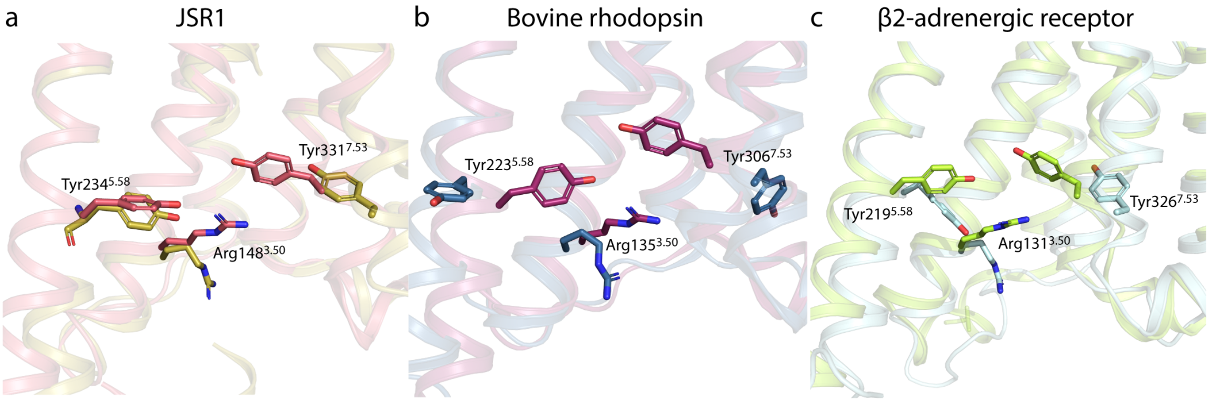

Supplementary Figure 13: Conformational changes of Arg$^{3.50}$, Tyr$^{5.58}$ and Tyr$^{7.53}$ upon receptor activation. a: Inactive JSR1 is shown in yellow and active JSR1 in salmon. Arg148$^{3.50}$, Tyr234$^{5.58}$ and Tyr331$^{7.53}$ are shown as sticks in the respective color. Oxygen atoms are shown in red and nitrogen atoms in blue. **b:** Inactive bovine rhodopsin is shown in blue and active bovine rhodopsin in purple. Arg135$^{3.50}$, Tyr223$^{5.58}$ and Tyr306$^{7.53}$ are shown as sticks in the respective color. Oxygen atoms are shown in red and nitrogen atoms in blue. **c:** Inactive β2-adrenergic receptor is shown in pale cyan and active β2-adrenergic receptor is shown in pale green. Arg131$^{3.50}$, Tyr219$^{5.58}$ and Tyr326$^{7.53}$ are shown as sticks in the respective color. Oxygen atoms are shown in red and nitrogen atoms in blue.

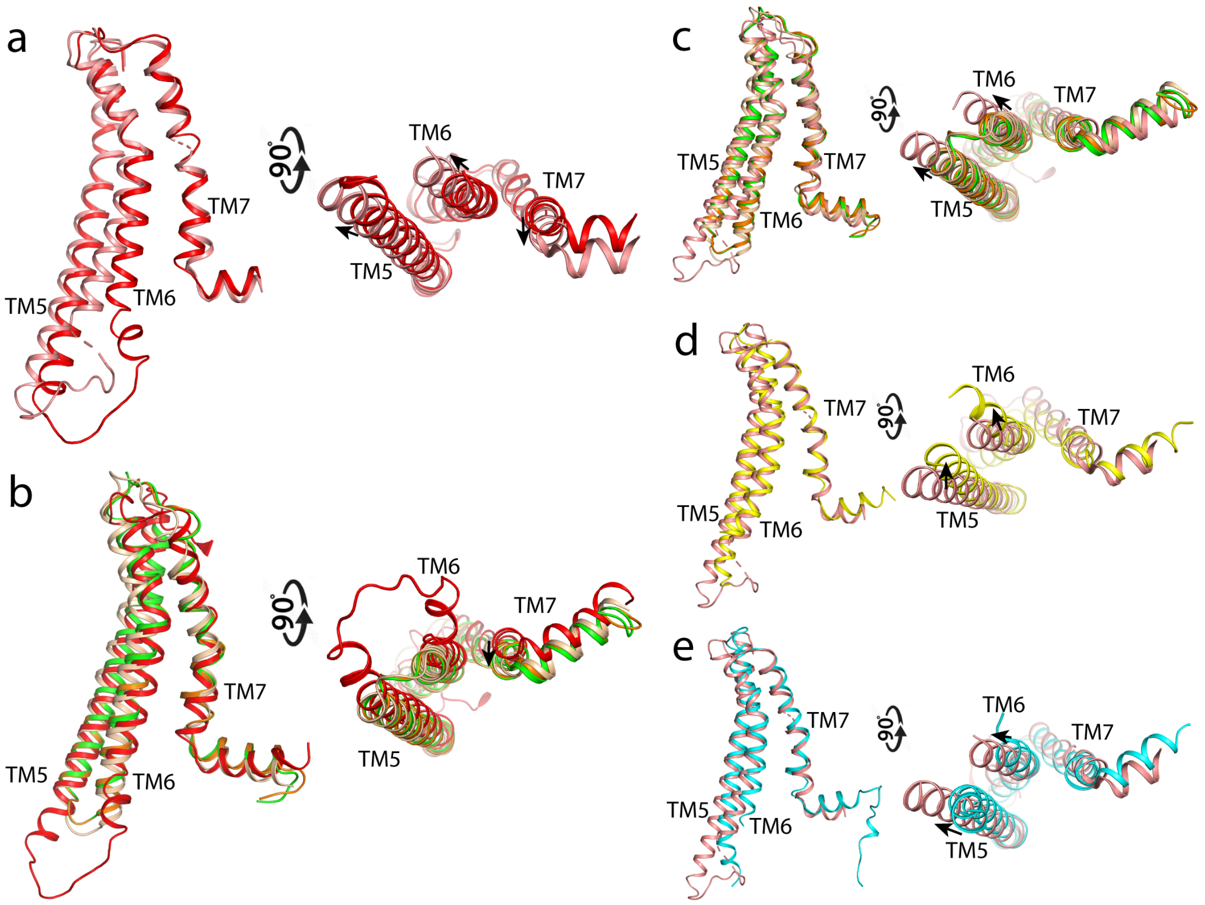

Supplementary Figure 14: TM5/6 and TM7 relocation comparison. a: Overlay of TM5/6/7 of the JSR1-jsG$_{iq}$_1 complex (salmon) and the JSR1-hG$_i$ complex (red). **b:** Overlay of TM5/6/7 of the JSR1-hG$_i$ complex with bovine metarhodopsin II bound to GαCT (PDB 3PQR (orange); PDB 4A4M (green)) and the rhodopsin-hG$_i$ complex (PDB 6CMO (wheat)). **c:** Overlay of TM5/6/7 of the JSR1-jsG$_{iq}$_1 complex with bovine metarhodopsin II bound to GαCT (PDB 3PQR (orange); PDB 4A4M (green)) and the rhodopsin-hG$_i$ structure (PDB 6CMO (wheat)). **d:** Overlay of TM5/6/7 of the JSR1-jsG$_{iq}$_1 complex (salmon) with the β2-adrenergic receptor-G$_s$ complex (PDB 3SN6 (yellow)). **e:** Overlay of TM5/6/7 of the JSR1-jsG$_{iq}$_1 complex (salmon) with the M1 receptor-G$_{11}$ complex (PDB 6OIJ (cyan)).

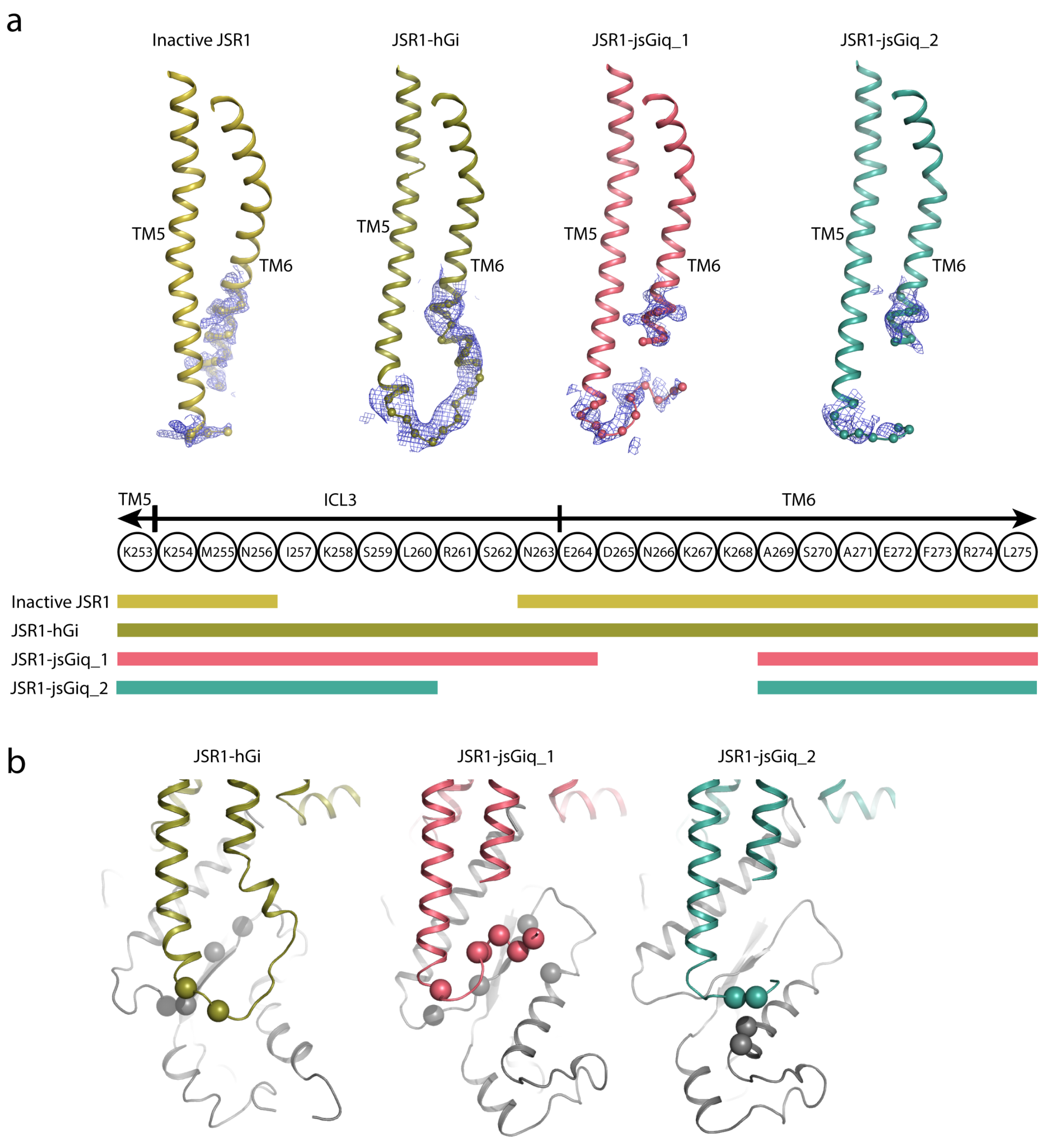

Supplementary Figure 15: Comparison of TM5-ICL3-TM6 in the JSR1 structures. a: The top panel shows structures of TM5-ICL3-TM6 of inactive JSR1 (PDB 6I9K), active JSR1 from JSR1-hG_i, active JSR1 from JSR1-jsG_{iq}_1 and active JSR1 from JSR1-jsG_{iq}_2. The experimental cryo-EM maps are shown for residues 253-275 of JSR1. The bottom panel shows a schematic representation of the residues 253-275. The colored rows show which residues are modeled (solid bar) and missing (no bar) in the respective structures. **b:** Observed contact between JSR1 ICL3 and the α4-β6 region of Gα. Based on the structural models, carbon α atoms of JSR1 ICL3 residues within 4 Å of the Gα α4-β6 region (and vice versa) are shown as spheres. JSR1 is colored in olive (JSR1-hG_i), salmon (JSR1-jsG_{iq}_1) and teal (JSR1-jsG_{iq}_2), respectively. Gα is colored in gray.

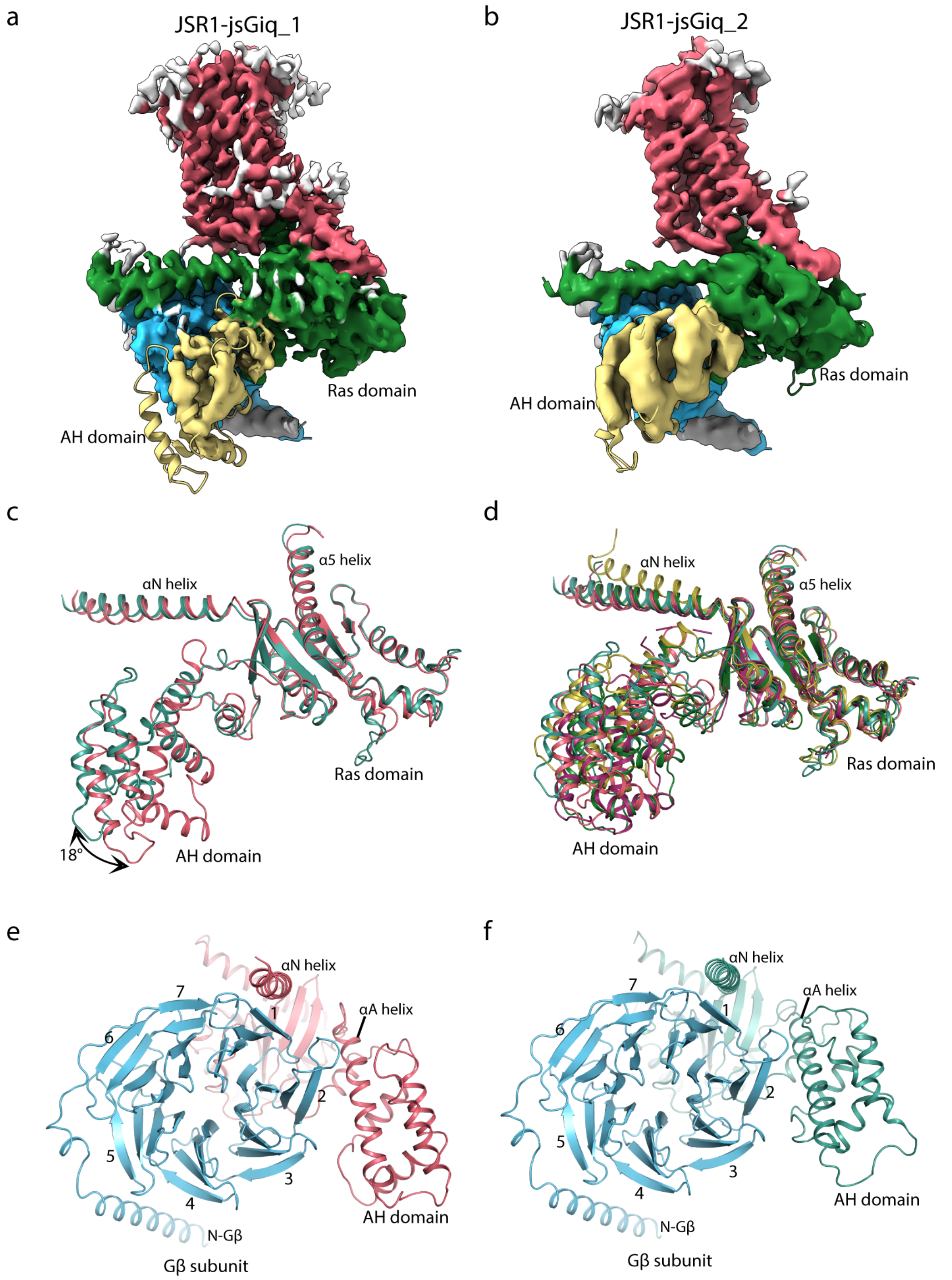

Supplementary Figure 16. a, b: Experimental cryo-EM maps of the JSR1-jsG$_{iq}$_1 complex (A) and JSR1-jsG$_{iq}$_2 (B) overlaid with their models. Specific colors are assigned for JSR1 (salmon), Gα Ras domain (green), Gα AH domain (yellow), Gβ (cyan) and Gγ (gray). **c:** Structural comparison of the jsGα$_{iq}$ subunit from JSR1-jsG$_{iq}$_1 (salmon) and JSR1-jsG$_{iq}$_2 (teal). The α-helical domain, αN helix, α5 helix and the Ras domain are labelled. **d:** Structural comparison of the Gα subunit from the JSR1-jsG$_{iq}$_1 (salmon), JSR1-jsG$_{iq}$_2 (teal), GABA(B) receptor-hG$_i$ (PDB 7EB2; yellow), NTSR1-hG$_i$ (PDB 7L0S; purple) and the cannabinoid receptor 2-hG$_i$ (PDB 6PT0; green) complex structures. The AH domain, αN helix, α5 helix and the Ras domain are labelled. **e:** Structure of the jsGα$_{iq}$ and Gβ subunits from JSR1-jsG$_{iq}$_1. The Gα subunit is colored in salmon and the Gβ subunit in cyan. The AH domain, αN helix and the αA helix of the jsGα$_{iq}$ are labelled. The numbers of the Gβ subunit represent the numbering of the β blades according to Wall *et al.* [1]. **f:** Structure of the jsGα$_{iq}$ and Gβ subunits from

JSR1-jsG$_{iq}$_2. The Gα subunit is colored in teal and the Gβ subunit in cyan. The AH domain, αN helix and the αA helix of the jsGα$_{iq}$ are labelled.

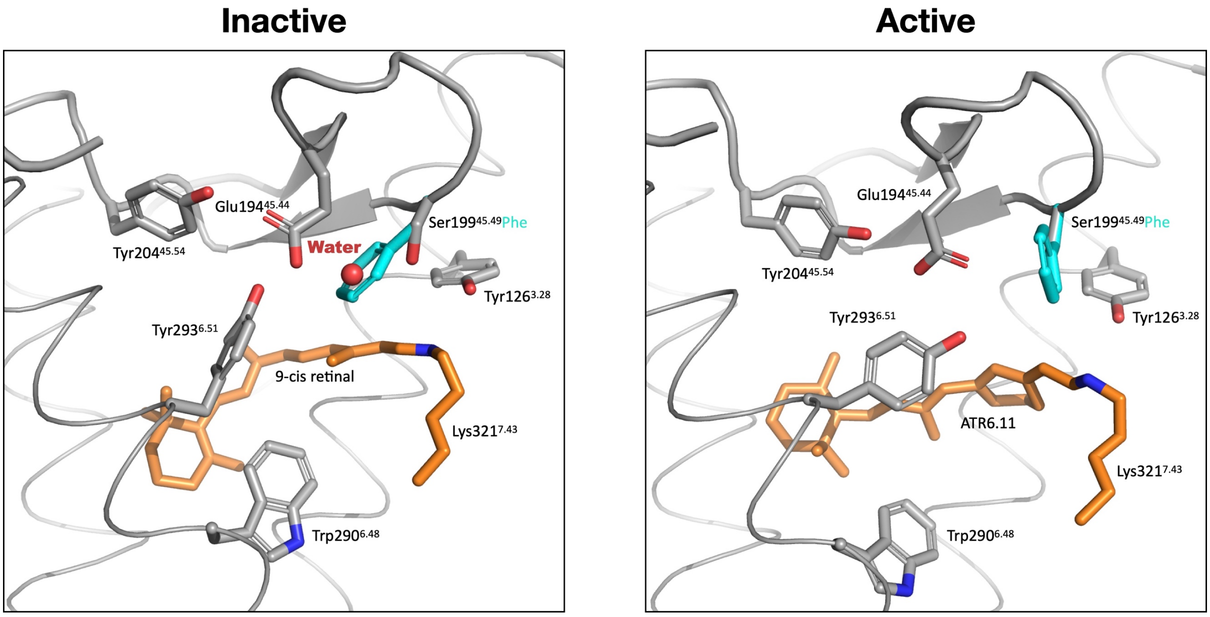

Supplementary Figure 17. Ser199$^{45.49}$ conformation modeled in inactive and active JSR1. In the inactive state, the Schiff base of the Ser199$^{45.49}$Phe mutant is deprotonated because the bulky aromatic phenylalanine most likely displaces the water molecule that is critical for the hydrogen bond network between the counterion Glu194$^{45.44}$ and the Schiff base. In the active-state JSR1, Ser199$^{45.49}$ changes its rotamer position, if such a change also occurs in Ser199$^{45.49}$Phe, the phenylalanine side chain would no longer interfere with the hydrogen bond network between Glu194$^{45.44}$ and the Schiff base. The proposed Ser199$^{45.49}$Phe side chain is shown in cyan. Inactive state JSR1 is represented by PDB 6I9K. The active state JSR1 is adapted from JSR1-jsG$_{iq}$_1.

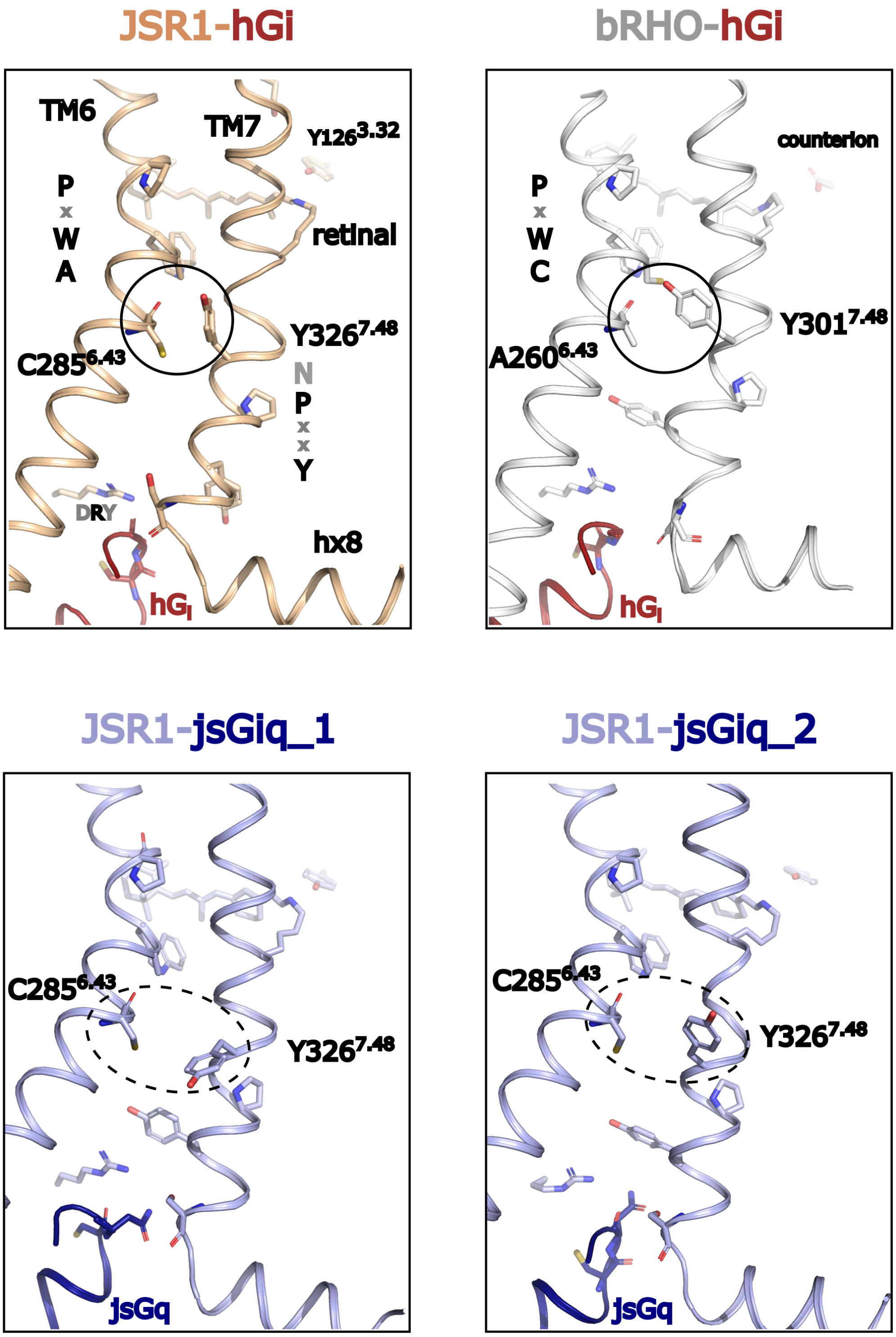

Supplementary Figure 18. Detail of TM6/TM7/H8 in the JSR1-hG$_i$ complex (top left), the two conformations of the JSR1-jsG$_{iq}$ complex (bottom), and the bovine rhodopsin-hG$_i$ complex (top right, PDB 6QNO). Top panels: The packing at the 6.43/7.48 residues (circled) is located right between the A/C-W-x-P motif in TM6 and the N-P-x-x-Y motif in TM7, and is similar in JSR1 and rhodopsin bound to hG$_i$. In bovine rhodopsin, the packing is stronger, including an inter-helical hydrogen bond. Bottom panels: This packing is looser in jsG$_{iq}$-bound JSR1.

	JSR1-jsG$_{iq}$_1 (EMDB-19882) (PDB 9EPP)	JSR1-jsG$_{iq}$_2 (EMDB-19883) (PDB 9EPQ)	JSR1-hG$_i$ (EMDB-19884) (PDB 9EPR)
**Data collection and processing**			
Magnification	165'000	165'000	105'000
Voltage (kV)	300	300	300
Electron exposure (e–/Å^2)	70	70	50
Defocus range (μm)	-1.0 to -2.4	-1.0 to -2.4	-0.7 to -2.4
Pixel size (Å)	0.51	0.51	0.85
Symmetry imposed	C1	C1	C1
Initial particle images (no.)	2'964'739	2'964'739	8'400'000
Final particle images (no.)	159'665	134'167	400'000
Map resolution (Å)	4.06	4.17	4.9
FSC threshold	0.143	0.143	0.143
Map resolution range (Å)	n.a.	n.a.	4.6 – 9.0
**Refinement**			
Initial model used (PDB code)	9EPP	9EPQ	9EPR
Model resolution (Å)	4.3	4.4	6.2
FSC threshold	0.5	0.5	0.5
Model resolution range (Å)	n.a.	n.a.	n.a.
Map sharpening *B* factor (Å^2)	-162	-206	-229
Model composition			
Non-hydrogen atoms	7482	7489	7046
Protein residues	946	948	890
Ligands	ATR611	ATR611	ATR
B factors (Å^2)			
Protein	76.12	83.33	169.94
Ligand	59.22	41.42	n.a.
R.m.s. deviations			
Bond lengths (Å)	0.004	0.004	0.006
Bond angles (°)	0.718	0.756	1.172
Validation			
MolProbity score	2.10	2.27	2.99
Clashscore	14.16	19.96	12.84
Poor rotamers (%)	1.47	1.72	14.43
Ramachandran plot			
Favored (%)	95.49	95.73	91.87
Allowed (%)	4.40	4.17	7.67
Disallowed (%)	0.11	0.11	0.46

Supplementary Table 1: Cryo-EM data collection, refinement and validation statistics

Residue	R148 (3.50)	V151 (3.53)	I152 (3.54)	M156 (34.51)	A157 (34.52)	P160 (34.55)	I237 (5.61)	H244 (5.68)	L248 (5.72)	Q251 (5.75)	K254 (5.78)	M255 (5.79)	L260 (ICL3)	R261 (ICL3)	S262 (ICL3)	N263 (ICL3)	R274 (6.32)	L275 (6.33)	V278 (6.36)	S334 (7.56)	H335 (8.47)	P336 (8.48)	K337 (8.49)
R31 (G.hns1.02)				orange	green																		
E308 (G.H4.16)															green								
E318 (G.h4s6.12)														orange		green							
I319 (G.h4s6.13)															green								
H322 (G.S6.04)												green											
C325 (G.s6h5.02)												green											
Q333 (G.H5.5)										orange	green												
F334 (G.H5.6)													green										
C337 (G.H5.9)										green													
K340 (G.H5.12)								green															
D341 (G.H5.13)								green						orange									
N347 (G.H5.19)			green																				
L348 (G.H5.20)			green																				
K349 (G.H5.21)																						green	
E350 (G.H5.22)																							orange
C351 (G.H5.23)	orange	green																					
N352 (G.H5.24)	green																			orange	orange	green	
L353 (G.H5.25)	green						green											green	green				
V354 (G.H5.26)																	orange		green				

Supplementary Table 2: JSR1-jsG$_{iq}$_1 interaction table. Top row shows the JSR1 residue number and GPCR numbering. Left column shows the jsG$_{iq}$ residue number and G protein numbering. Orange fields symbolize polar interactions and green fields residues within 4 Å distance.

**Residue**	R148 (3.50)	V151 (3.53)	I152 (3.54)	M156 (34.51)	A157 (34.52)	P160 (34.55)	H244 (5.68)	L248 (5.72)	Q251 (5.75)	K254 (5.78)	M255 (5.79)	I257 (ICL3)	K258 (ICL3)	L260 (ICL3)	R274 (6.32)	V278 (6.36)	Y331 (7.53)	S334 (7.56)	H335 (8.47)	P336 (8.48)	K337 (8.49)
R31 (G.hns1.02)																					
I194 (G.S3.01)																					
E298 (G.H4.05)																					
A301 (G.H4.08)																					
C337 (G.H5.9)																					
K340 (G.H5.12)																					
D341 (G.H5.13)																					
I343 (G.H5.15)																					
K349 (G.H5.21)																					
E350 (G.H5.22)																					
N352 (G.H5.24)																					
L353 (G.H5.25)																					
V354 (G.H5.26)																					

Supplementary Table 3: JSR1-jsG$_{iq}$_2 interaction table. Top row shows the JSR1 residue number and GPCR numbering. Left column shows the jsG$_{iq}$ residue number and G protein numbering. Orange fields symbolize polar interactions and green fields residues within 4 Å distance.

Protein	Sequence	Sources
JSR1	MLPHAAKMAARVAGDHDGRNISIVDLLPEDMLPMIHEHWYKFPPME<br>TSMHYILGMLIIVIGIISVSGNGVVMYLMMTVKNLRTPGNFLVLNL<br>ALSDFGMLFFMMPTMSINCFAETWVIGPFMCELYGMIGSLFGSASI<br>WSLVMITLDRYNVIVKGMAGKPLTKVGALLRMLFVWIWSLGWTIAP<br>MYGWSRYVPEGSMTSCTIDYIDTAINPMSYLIAYAIFVYFVPLFII<br>IYCYAFIVMQVAAHEKSLREQAKKMNIKSLRSNEDNKKASAEFRLA<br>KVAFMTICCWFMAWTPYLTLSFLGIFSDRTWLTPMTSVWGAIFAKA<br>SACYNPIVYGISHPKYRAALHDKFPCLKCGSDSPKGDSASTVAESE<br>KAGEETSQVAPA	Expression in GnTI-deficient HEK293
Human Gαi1	MKKHHHHHHHHHHENLYFQGGSMGCTLSAEDKAAVERSKMIDRNLR<br>EDGEKAAREVKLLLLGAGESGKSTIVKQMKIIHEAGYSEEECKQYK<br>AVVYSNTIQSIIAIIRAMGRLKIDFGDSARADDARQLFVLAGAAEE<br>GFMTAELAGVIKRLWKDSGVQACFNRSREYQLNDSAAYYLNDLDRI<br>AQPNYIPTQQDVLRTRVKTTGIVETHFTFKDLHFKMFDVGGQRSER<br>KKWIHCFEGVTAIIFCVALSDYDLVLAEDEEMNRMHESMKLFDSIC<br>NNKWFTDTSIILFLNKKDLFEEKIKKSPLTICYPEYAGSNTYEEAA<br>AYIQCQFEDLNKRKDTKEIYTHFTCATDTKNVQFVFDAVTDVIIKN<br>NLKDCGLF	Expression in E. coli
jsGαiq	MGCTLSAEDKAAVERSKMIDRNLREDGEKARREVKLLLLGAGESGK<br>STIVKQMKIIHEAGYSEEECKQYKAVVYSNTIQSIIAIIRAMGRLK<br>IDFGDSARADDARQLFVLAGAAEEGFMTAELAGVIKRLWKDSGVQA<br>CFNRSREYQLNDSAAYYLNDLDRIAQPNYIPTQQDVLRTRVKTTGI<br>VETHFTFKSIHFKMFDVGGQRSERKKWIHCFEGVTAIIFCVALSDY<br>DLVLAEDEEMNRMHESMKLFDSICNNKWFTDTSIILFLNKKDLFEE<br>KIKKSPLTICYPEYAGSNTYEEAAAYIQCQFEDLNKRKDTKEIYTH<br>FTCATDTKNVQFVFCAVKDTILQNNLKECNLV	Expression in Hi5
Bovine Gβ1	MSELDQLRQEAEQLKNQIRDARKACADATLSQITNNIDPVGRIQMR<br>TRRTLRGHLAKIYAMHWGTDSRLLVSASQDGKLIIWDSYTTNKVHA<br>IPLRSSWVMTCAYAPSGNYVACGGLDNICSIYNLKTREGNVRVSRE<br>LAGHTGYLSCCRFLDDNQIVTSSGDTTCALWDIETGQQTTTFTGHT<br>GDVMSLSLAPDTRLFVSGACDASAKLWDVREGMCRQTFTGHESDIN<br>AICFFPNGNAFATGSDDATCRLFDLRADQELMTYSHDNIICGITSV<br>SFSKSGRLLLAGYDDFNCNVWDALKADRAGVLAGHDNRVSCLGVTD<br>DGMAVATGSWDSFLKIWN	Extraction from Bovine retina
Human Gβ1	MHHHHHHHHHHLEVLFQGPGSSGSELDQLRQEAEQLKNQIRDARKA<br>CADATLSQITNNIDPVGRIQMRTRRTLRGHLAKIYAMHWGTDSRLL<br>VSASQDGKLIIWDSYTTNKVHAIPLRSSWVMTCAYAPSGNYVACGG<br>LDNICSIYNLKTREGNVRVSRELAGHTGYLSCCRFLDDNQIVTSSG<br>DTTCALWDIETGQQTTTFTGHTGDVMSLSLAPDTRLFVSGACDASA<br>KLWDVREGMCRQTFTGHESDINAICFFPNGNAFATGSDDATCRLFD<br>LRADQELMTYSHDNIICGITSVSFSKSGRLLLAGYDDFNCNVWDAL<br>KADRAGVLAGHDNRVSCLGVTDDGMAVATGSWDSFLKIWN	Expression in Hi5
Bovine Gγ1	MPVINIEDLTEKDKLKMEVDQLKKEVTLERMLVSKCCEEFRDYVEE<br>RSGEDPLVKGIPEDKNPFKELKGGCVIS	Extraction from Bovine retina
Human Gγ2	MASNNTASIAQARKLVEQLKMEANIDRIKVSKAAADLMAYCEAHAK<br>EDPLLTPVPASENPFREKKFFCAIL	Expression in Hi5

Supplementary Table 4: Sequences and production sources of JSR1 and G protein subunits.

1 Wall, M. A. *et al.* The structure of the G protein heterotrimer Gi alpha 1 beta 1 gamma 2. *Cell* **83**, 1047-1058 (1995).
