# Supplementary material for: Active state structures of a bistable visual opsin bound to G proteins
Source: Nat Commun. 2024 Oct 16;15:8928. doi: 10.1038/s41467-024-53208-2 (PMC11484933; doi:10.1038/s41467-024-53208-2)
Supplement: Supplementary file 2 — Description of Additional Supplementary Files [file 41467_2024_53208_MOESM2_ESM.pdf]

Description of Additional Supplementary Files

Supplementary Movie 1:

Local resolution map of JSR1-jsG$_{iq}$_1 complex. The electron potential map is colored based on the local resolution values (Local Resolution Estimation, cryoSPARC).

Supplementary Movie 2:

3D single particle variability analysis of the JSR1-jsG$_{iq}$ complex. Particles from JSR1-jsG$_{iq}$_1 and JSR1-jsG$_{iq}$_2 were combined for the 3D variability analysis (cryoSPARC). Cryo-EM map is shown from three different views.

Supplementary Movie 3:

Retinal binding pocket of the inactive state JSR1. Model of the inactive state JSR1 bound to 9-cis retinal (PDB: 6I9K) shown in yellow and cartoon representation. 9-cis retinal and Lys321 are shown in orange in sphere representation. Residues within 4 Å of the 9-cis retinal are shown in yellow in sphere representation.

Supplementary Movie 4:

Retinal binding pocket of the active state JSR1. Model of the active JSR1 bound to ATR6.11 shown in salmon color and cartoon representation. ATR6.11 and Lys321 are shown in yellow in sphere representation. Residues within 4 Å of the ATR6.11 are shown in salmon in sphere representation.

Supplementary Movie 5:

Retinal binding pocket of the inactive state bovine rhodopsin. Model of the inactive state bovine rhodopsin bound to 11-cis retinal (PDB: 1GZM) shown in orange and cartoon representation. 11-cis retinal and Lys296 are shown in yellow and sphere representation. Residues within 4 Å of the 11-cis retinal are shown in orange and sphere representation.

Supplementary Movie 6:

Retinal binding pocket of the active state bovine rhodopsin. Model of the active state bovine rhodopsin bound to all-trans retinal (PDB:4A4M) shown in green and cartoon representation. All-trans retinal and Lys296 are shown in yellow and sphere representation. Residues within 4 Å of the all-trans retinal are shown in green and sphere representation.

Supplementary Movie 7:

Morph between the JSR1-jsG$_{iq}$_1 and JSR1-jsG$_{iq}$_2 models without the AHD. Models are shown in cartoon style.

Supplementary Movie 8:

Morph between the JSR1-jsG$_{iq}$_1 and JSR1-jsG$_{iq}$_2 models with the AHD. Models are shown in cartoon style.
