# Supplementary material for: Active state structures of a bistable visual opsin bound to G proteins
Source: Nat Commun. 2024 Oct 16;15:8928. doi: 10.1038/s41467-024-53208-2 (PMC11484933; doi:10.1038/s41467-024-53208-2)
Supplement: Supplementary file 11 — Reporting Summary [file 41467_2024_53208_MOESM11_ESM.pdf]

nature portfolio

Corresponding author(s): Ching-Ju Tsai

Last updated by author(s): Sep 16, 2024

Reporting Summary

Nature Portfolio wishes to improve the reproducibility of the work that we publish. This form provides structure for consistency and transparency in reporting. For further information on Nature Portfolio policies, see our Editorial Policies and the Editorial Policy Checklist.

Statistics

For all statistical analyses, confirm that the following items are present in the figure legend, table legend, main text, or Methods section.

n/a	Confirmed	
☐	☒	The exact sample size (*n*) for each experimental group/condition, given as a discrete number and unit of measurement
☐	☒	A statement on whether measurements were taken from distinct samples or whether the same sample was measured repeatedly
☐	☒	The statistical test(s) used AND whether they are one- or two-sided<br>*Only common tests should be described solely by name; describe more complex techniques in the Methods section.*
☒	☐	A description of all covariates tested
☒	☐	A description of any assumptions or corrections, such as tests of normality and adjustment for multiple comparisons
☐	☒	A full description of the statistical parameters including central tendency (e.g. means) or other basic estimates (e.g. regression coefficient) AND variation (e.g. standard deviation) or associated estimates of uncertainty (e.g. confidence intervals)
☒	☐	For null hypothesis testing, the test statistic (e.g. *F*, *t*, *r*) with confidence intervals, effect sizes, degrees of freedom and *P* value noted<br>*Give P values as exact values whenever suitable.*
☒	☐	For Bayesian analysis, information on the choice of priors and Markov chain Monte Carlo settings
☒	☐	For hierarchical and complex designs, identification of the appropriate level for tests and full reporting of outcomes
☒	☐	Estimates of effect sizes (e.g. Cohen's *d*, Pearson's *r*), indicating how they were calculated

Our web collection on statistics for biologists contains articles on many of the points above.

Software and code

Policy information about availability of computer code

Data collection	Cryo-EM data was collected automatically using EPU.
Data analysis	cryoSPARC (v4.0), Relion (v3.1), CTFFIND4.1, cyYOLO (v1.6), SIDESPLITTER, MotionCor2, Patch CTF Estimation, UCSF ChimeraX (v1.7), Pymol (v2.5.5), Phenix (v1.20-1), Coot (v0.9.8.92).

For manuscripts utilizing custom algorithms or software that are central to the research but not yet described in published literature, software must be made available to editors and reviewers. We strongly encourage code deposition in a community repository (e.g. GitHub). See the Nature Portfolio guidelines for submitting code & software for further information.

Data

Policy information about availability of data

All manuscripts must include a data availability statement. This statement should provide the following information, where applicable:

- Accession codes, unique identifiers, or web links for publicly available datasets
- A description of any restrictions on data availability
- For clinical datasets or third party data, please ensure that the statement adheres to our policy

Atomic coordinates of JSR1-jsGiq_1, JSR1-jsGiq_2 and JSR1-hGi without the AHD are dasdeposited to the Protein Data Bank under accession codes 9EPP, 9EPQ and 9EPR, respectively. Cryo-EM maps of JSR1-jsGiq_1, JSR1-jsGiq_2 and JSR1-hGi are deposited in the Electron Microscopy Data Bank under accession code EMD-19883, EMD-19884 and EMD-19884, respectively.

April 2023

Research involving human participants, their data, or biological material

Policy information about studies with human participants or human data. See also policy information about sex, gender (identity/presentation), and sexual orientation and race, ethnicity and racism.

Reporting on sex and gender	N/A
Reporting on race, ethnicity, or other socially relevant groupings	N/A
Population characteristics	N/A
Recruitment	N/A
Ethics oversight	N/A

Note that full information on the approval of the study protocol must also be provided in the manuscript.

Field-specific reporting

Please select the one below that is the best fit for your research. If you are not sure, read the appropriate sections before making your selection.

[x] Life sciences [ ] Behavioural & social sciences [ ] Ecological, evolutionary & environmental sciences

For a reference copy of the document with all sections, see nature.com/documents/nr-reporting-summary-flat.pdf

Life sciences study design

All studies must disclose on these points even when the disclosure is negative.

Sample size	Sample sizes were not predetermined by statistical methods. Cryo-EM data size was determined by the accessibility of the microscope. These data sizes were sufficient for 3D reconstruction of the map of the protein complexes. Glo-assay samples were octuplicates.
Data exclusions	Cryo-EM data images were omitted when their CTF-fit resolution was worse than 8 Angstrom. Glo-assay outliers were identified using the Grubbs-test and therefore omitted.
Replication	Glo-assay samples were octuplicates.
Randomization	No randomization was necessary.
Blinding	No blinding was necessary.

Reporting for specific materials, systems and methods

We require information from authors about some types of materials, experimental systems and methods used in many studies. Here, indicate whether each material, system or method listed is relevant to your study. If you are not sure if a list item applies to your research, read the appropriate section before selecting a response.

Materials & experimental systems

n/a	Involved in the study	
[ ]	[x]	Antibodies
[ ]	[x]	Eukaryotic cell lines
[x]	[ ]	Palaeontology and archaeology
[x]	[ ]	Animals and other organisms
[x]	[ ]	Clinical data
[x]	[ ]	Dual use research of concern
[x]	[ ]	Plants

Methods

n/a	Involved in the study	
[x]	[ ]	ChIP-seq
[x]	[ ]	Flow cytometry
[x]	[ ]	MRI-based neuroimaging

Antibodies

Antibodies used	1D4 antibody

Validation	This antibody targets the C-terminal amino acid sequence TETSQVAPA, which is also termed 1D4 tag. This sequence originates from the C-terminus of animal Rhodopsin (human and bovine). The antibody has been developed as an affinity purification tool for proteins engineered to contain a C-terminal 1D4 sequence.

Eukaryotic cell lines

Policy information about cell lines and Sex and Gender in Research

Cell line source(s)	HEK293 (GnTI-deficient), Spodoptera frugiprtda cell line sf9, High Five insect cells.
Authentication	Cells are commercially purchased.
Mycoplasma contamination	Mycoplasma contamination not tested.
Commonly misidentified lines (See ICLAC register)	No commonly misidentified lines used.

Plants

Seed stocks	N/A
Novel plant genotypes	N/A
Authentication	N/A
